# Susceptibility to Re-Vaccination

**Published:** 1902-01-18

**Authors:** 


### SUSCEPTIBILITY TO RE-VACCINATION.

Dr. Andrewes[1] publishes an interesting analysis of the results observed on re-vaccinating the female staff at St. Bartholomew's Hospital, including sisters, nurses, wardmaids, and servants. His figures relate to 158 cases vaccinated by the same method, with lymph from the same source, and, as he believes, of very uniform potency. He classifies the results under three heads. (1) Perfect vaccinia—*i.e.*, large well-formed vesicles resembling those of primary vaccinia in all respects save that the process was often accelerated by a day or two; (2) imperfect vaccinia—vesicles small, imperfect, and generally much accelerated; and (3) negative result—transitory redness without the formation of any vesicles. The gross result worked out as follows:—Perfect, 45·57 per cent.; imperfect, 41·14 per cent.; negative, or 12·29 per cent. Taking, however, the different classes, 55 were wardmaids and domestic servants—young adults who for the most part had not been vaccinated since infancy. Of these 76·36 per cent. had perfect vaccinia, 20 per cent. imperfect, and 3·64 per cent. negative result. Among the 88 nurses, who are obliged to be re-vaccinated before entering upon their hospital training, although some of whom had managed to evade the regulation and in others the re-vaccination had been unsuccessful, the results were very different, only 27·27 per cent. had perfect vaccinia, 54·54 per cent. had imperfect, and 18·18 per cent. negative results. The sisters and senior nurses occupied an intermediate position. It should be added that except for special reasons, such as known exposure to small-pox infection, no attempt was made to re-vaccinate any person who had been successfully re-vaccinated within five years. Commenting on the results obtained by a careful analysis of the various periods which had elapsed since the last asserted successful re vaccination, Dr. Andrewes says that the well-known fact that the immunity against small-pox which vaccination confers is by no means permanent is a strong argument for undergoing efficient re-vaccination. It is of course an open question how far susceptibility to re-vaccination implies susceptibility to small-pox. Nevertheless, re-vaccination is the only practical test at our command for determining susceptibility to the major disease, and we must accept immunity against properly applied vaccinia performed vaccination as a fair proof of immunity against small-pox. When small-pox is present one is constantly asked "ought I to be re-vaccinated?" and according to Dr. Andrewes, the only answer to this question is "Be re-vaccinated and see. If you do not need it, it will not take, and you will suffer no inconvenience; if you need it you will be compensated for your inconvenience."

[1] Lancet, Jan. 11.

